# Novel chitosan-polycaprolactone blends as potential scaffold and carrier for corneal endothelial transplantation

**Published:** 2012-01-31

**Authors:** Tsung-Jen Wang, I-Jong Wang, Jui-Nan Lu, Tai-Horng Young

**Affiliations:** 1 Department of Ophthalmology, Taipei Medical University Hospital, Taipei, Taiwan; 2 Department of Ophthalmology, School of Medicine, College of Medicine, Taipei Medical University, Taipei, Taiwan; 3 Institute of Biomedical Engineering, College of Medicine and College of Engineering, National Taiwan University, Taipei, Taiwan; 4 Department of Ophthalmology, National Taiwan University Hospital and College of Medicine, Taipei, Taiwan

## Abstract

**Purpose:**

The aim of this prospective study was to evaluate whether blending two kinds of biomaterials, chitosan and polycaprolactone (PCL), can be used as scaffold and carrier for growth and differentiation of corneal endothelial cells (CECs).

**Methods:**

A transparent, biocompatible carrier with cultured CECs on scaffold would be a perfect replacement graft. In the initial part of experiment, for essential and biocompatible test, chitosan and PCL were evaluated respectively and blended in various proportions by coating. In the later part of this study, for evaluation of potential application, homogenous solutions of 25%, 50%, and 75% PCL compositions were attempted to structure blend membranes.

**Results:**

Chitosan, PCL 25, PCL 50, and PCL 75 blends could maintain transparency of culturing substrata. BCECs were found to be reached confluence successfully after 7 days on PCL 25, PCL 50, and PCL 75. The expression of tight junction and extracellular matrix protein were observed as well. Alternatively, only PCL 25 could make blend membrane with enough strength during preparation for carrier in culture. On this blend membrane, the growth pattern and phenotype of BCECs could be observed well.

**Conclusions:**

A ratio of 75:25 (chitosan:PCL) blends showed enough mechanical properties as well as suitable support for cellular activity in cultivating BCECs. Thus, a novel methodology of biodegradable carrier from chitosan and PCL has potential to be a good replacement scaffold for raising CECs for clinical transplantation.

## Introduction

Corneal disease is the major cause of blindness. Over 10 million individuals experience corneal blindness worldwide [1]. Currently, corneal transplantation is the only treatment for restoring vision. Among the performed tissue transplants in the US, corneal grafts are the most common with more than 50,000 annually. Moreover, 150,000 or so corneal transplants are performed yearly in the world. Although the successful rate of total corneal transplantation is more than 90% at 1 year and 70% at 5 years, the penetrating keratoplasty for corneal endothelial dysfunction is not risk free [2]. The shortcomings of corneal transplantation include the possibilities of infections, immunological rejection (increase to 25% by 4–5 years), and most important of all, insufficient donor corneas [3]. Additionally, increased laser-assisted in situ keratomileusis (LASIK) surgeries have also diminished the supply of healthy donor tissue. Up to date, still millions of patients around the world have need of corneal transplantation.

The human corneal endothelial cells (HCECs) originate from neural crest and cover the posterior surface of cornea as a monolayer. They maintain corneal transparency by regulating corneal hydration through its pump and barrier functions [4]. When they loss due to dystrophy, trauma, or surgical intervention, a compensatory enlargement of the remaining cells followed and resulted in irreversible corneal endothelial dysfunction. In these situations, an alternative method for replacing the endothelium without corneal trephination and sutures can save the vision of patients. Posterior lamellar keratoplasty including deep lamellar endothelial keratoplasty (DLEK) [5,6], Descemet’s stripping endothelial keratoplasty (DSEK) [7], and Descemet’s stripping automated endothelial keratoplasty (DSAEK) [8] has been developed in recent years. The advantages of these operations are less suture-related graft complications, reduced astigmatism, better visual prognosis, and safer closed-eye surgery. However, the supply of donor cornea remains uncertain and usually makes patients wait for a long time.

Within the anterior chamber, HCECs are immersed in aqueous humor, and cytokines such as transforming growth factor-beta2 (TGF-β2) were illustrated to inhibit proliferation of HCECs by hampering the G_1_-to-S transition [4]. Furthermore, contact-dependent inhibition also plays a major role in the induction of cell-cycle arrest [9]. Even if the human corneal endothelium is held in a non-proliferative state within the eye, HCECs do retain the ability to proliferate [4,9,10]. Accordingly, one promising opportunity for relieving the requirement for donated cornea is the development of a tissue-engineering corneal equivalent. Techniques for cultivating HCECs have been reported [11,12], and effort has been made to build up transplantation models of cultivated human HCEC sheets using carriers from either natural tissue materials [13,14] or artificial polymeric materials [15,16]. Though attempts had been made to develop transplantation models, there are still many problems including insufficient supply of donors for corneal graft, cell culture drawbacks, bio-toxicity, and biocompatibility. Therefore, new biomaterial selections are necessitated for the development of clinical demands for corneal replacements in regenerative medicine.

In the field of bioengineered corneal endothelium, discovering an ideal carrier material has been always an important issue. The perfect cell carriers for corneal endothelial transplantation should be cellular innocuously, biodegradable appropriately, and handle easily. This goal may not be approached by single biomaterial. Blending two polymers is a chance to develop novel biomaterials with combinations of individual properties. Chitosan and polycaprolactone (PCL) are biodegradable biomaterials approved by Food and Drug Administration (FDA) with numerous advantages respectively. Surprisingly, the biologic, degradation or mechanical properties of polymer blends have disclosed promising results in comparison to that on the separate culturing substratum [17,18]. Based on different characteristics of chitosan and PCL, the present study hypothesizes that it is possible to create a new blend material that can combine the features of chitosan and PCL concurrently to be a scaffold and carrier for CEC culture and transplantation.

## Methods

### Preparation of culturing substrates

In the first part of experiments, chitosan (degree of deacetylation=85%; Sigma-Aldrich, St. Louis, MO) was dissolved in 0.5 M acetic acid to prepare 1 wt% chitosan solutions. PCL (Sigma-Aldrich) was dissolved in glacial acetic acid to prepare 10 wt% PCL solutions. To obtain 25, 50 and 75 wt% PCL in chitosan/PCL solutions, different volume of 10 wt% PCL solutions and glacial acetic acid were slowly added to 3 ml of 1 wt% chitosan. For simplified expression, the substrates were namely chitosan, PCL 25, PCL 50, PCL75, and PCL 100 according to PCL proportion. Then culturing plates were prepared by coating polymer solution onto 6- or 24-well tissue cultured polystyrene (TCPS; Costar, Corning, NY) directly. After 30 min of incubation at room temperature, the solutions were removed and the wells were dried in a convection oven at 55 °C for 24 h. The prepared substrates were neutralized in 0.5 N NaOH aqueous solutions for 24 h and were then washed thoroughly with deionized water. Before cell culturing, the coated and uncoated wells were exposed to ultraviolet light overnight.

### Measurement of transparency and transmittance

Substrates were prepared in 60 mm TCPS (Costar) to determine transparency. The plates were then placed on the graph paper and transparency of the prepared substrates was photographed by a digital camera. The transparency was also quantified by determining light transmittance of the membrane by a home-built device. In brief, the initial illumination from a white light source was measured by a digital lux meter (MLM-1010; minipa®, Sao Paulo, Brazil). Initially, a blank TCPS plate, serving as the control, was inserted between the light source and the digital lux meter at 100% transmittance. After that, chitosan, PCL 25, PCL 50, PCL 75, and PCL 100 were inserted respectively and illumination was measured again. Transmittance ratio is defined as the value of the prepared substrates relative to that of the blank TCPS plate.

### Primary culture of BCECs

This animal study was performed in line with an animal use protocol approved by the Review Committee of Taipei Medical University. Fresh bovine eyes were acquired from the local abattoir and immersed in iodine solution for 3 min, then transferred into phosphate-buffered saline (PBS; Invitrogen, Carlsbad, CA). As a modification from previous studies [19,20], fresh bovine corneal endothelial sheets were peeled and digested by trypsin in 37 °C for 30 to 60 min. Next, endothelial cells were collected by centrifugation of the supernatant (750× g for 5 min). Cell culture was performed in the supplemented hormonal epithelial medium (SHEM), which is composed of equal volumes of HEPES-buffered DMEM and Ham F12 (Invitrogen) supplemented with 5% FBS, 0.5% dimethyl sulfoxide (Sigma-Aldrich), 2 ng/ml hEGF (Sigma-Aldrich), 5 ug/ml insulin, 5 ug/ml transferrin, 5 ng/ml selenium (Invitrogen), 1 nM cholera toxin (Sigma-Aldrich), 50 μg/ml gentamicin (Invitrogen), and 1.25 μg/ml amphotericin B (Invitrogen). The flask was incubated at 37 °C in an atmosphere of 95% air/5% CO_2_. When cells reached confluence after 14–21 days, they were rinsed in PBS, detached by trypsinization with 0.05% trypsin for 5 min at 37 °C, and spun down. The cells were then suspended into SHEM. Subsequently, about 50,000 cells in each 24-well culturing plate were loaded and maintained in a humidified atmosphere with 5% CO_2_ at 37 °C. The media was changed every 2 to 3 days for up to 7 days in SHEM. The morphology of cells was viewed under a phase contrast microscope (Leica, Wetzlar, Germany).

### Immunohistochemistry

BCECs on culturing substrates and blend membranes were fixed at indicated time points by 4% fresh buffered paraformaldehyde (pH 7.4) at room temperature for 30 min. After being blocked with 10% BSA containing 0.5% Triton-X at room temperature for 1 h, cells were incubated overnight at 4 °C with a 1:200 dilution of anti-ZO-1 (Millipore, Temecula, CA). After washing twice with PBS for 15 min, samples were incubated with a 1:100 dilution of Alexa Fluor 568-conjugated secondary antibodies (Invitrogen) at room temperature for 1 h. For observing actin cytoskeleton BCECs on PCL 25 membrane, Alexa-Fluor phalloidin 546 secondary antibodies were used to stain cytoskeletal filamentous actin. All samples were stained with DAPI (1:5000; Invitrogen) at room temperature for 5 min. After several washes, all samples were mounted in fluorescent mounting solution (VectA Mount; Vector Laboratories, Burlingame, CA). Immunofluorescent images were obtained using Zeiss Axiovert 200 inverted microscope (Carl Zeiss, Jena, Germany) or Leica TCS SP5 confocal spectral microscope imaging system (Leica).

### Western blot analysis

Western blot analysis for collagen type IV expression of BCECs after being cultured on different substrates for 7 days was performed in standard protocols. In brief, proteins from cell extracts were separated by sodium dodecyl sulfate PAGE (SDS–PAGE) on 8% Tris-HCl reducing gels, transferred to PVDF membrane (Bio-Rad, Hercules, CA), and blocked with blocking solution overnight. Collagen type IV expression was detected by using goat antibody against collagen type IV (1:1000; Millipore) or mouse anti-β-actin (1:10,000; Invitrogen), followed by appropriate secondary antibodies (rabbit anti-goat, 1:10,000; Santa Cruz Biotechnology, Inc., Santa Cruz, CA) conjugated with horseradish peroxidase. Immunoreactive bands were visualized with an enhanced chemiluminescence (ECL; Perkin-Elmer Life Science, Boston, MA) system. The results were also presented as the ratio of collagen type IV expression of BCECs on chitosan/ PCL blends relative to that on chitosan.

### Preparation of chitosan and PCL blend membranes

In the second part of the experiments, chitosan and PCL blend membranes were prepared by mixing 75:25 (PCL 25), 50:50 (PCL 50), 25:75 (PCL 75; wt% of chitosan with wt% of PCL), respectively. The mixture of chitosan and PCL polymer was dissolved by formic acid in 5 wt%. Polymer solutions were coating on glass plate, and then evaporated in a convection oven at 55 °C over 24 h. The nascent membranes on glass plate were placed in 0.5 N NaOH until the membranes detached from the glass plate. Prevented from splitting and dryness, prepared membranes were then placed in 10 cm glass plate with PBS. The plates were then placed with black background and photographed by a digital camera. However, when preparing for cell culture, only pure chitosan and PCL 25 (75 wt% of chitosan with 25 wt% of PCL) can make membranes with enough strength successfully. The PCL 50 and PCL 75 membranes were fragile easily. Subsequently, the residual solvent in the chitosan and PCL 25 membranes was removed by a series of washing steps. Finally, those membranes were cut in size to fit 6- or 24-well TCPS, and sterilized with 70% alcohol under ultraviolet light overnight and then rinsed extensively with PBS before usage. Chitosan and PCL 25 membrane in 6-well TCPS size placed on the vision screen chart and transparency of the prepared membranes was photographed by a digital camera.

### Seeding of BCECs on PCL 25 membrane

Passage 2–3 confluent, subcultured cells derived from fresh bovine eyes were seeded onto PCL 25 membrane. The seeded cell numbers was 50 000, and the cells were cultivated for 7 days.

### Statistical analysis

All experiments were repeated at least three times from different groups. Statistical analyses were performed with SPSS software (SPSS Inc., Chicago, IL). Means were reported alongside with standard deviation. Comparative analysis of the results was conducted using a two-tailed *t* test for western blot analysis. A p value of <0.05 or 0.01 indicated statistical significance.

## Results

### Transparency and transmittance

**Figure 1:**
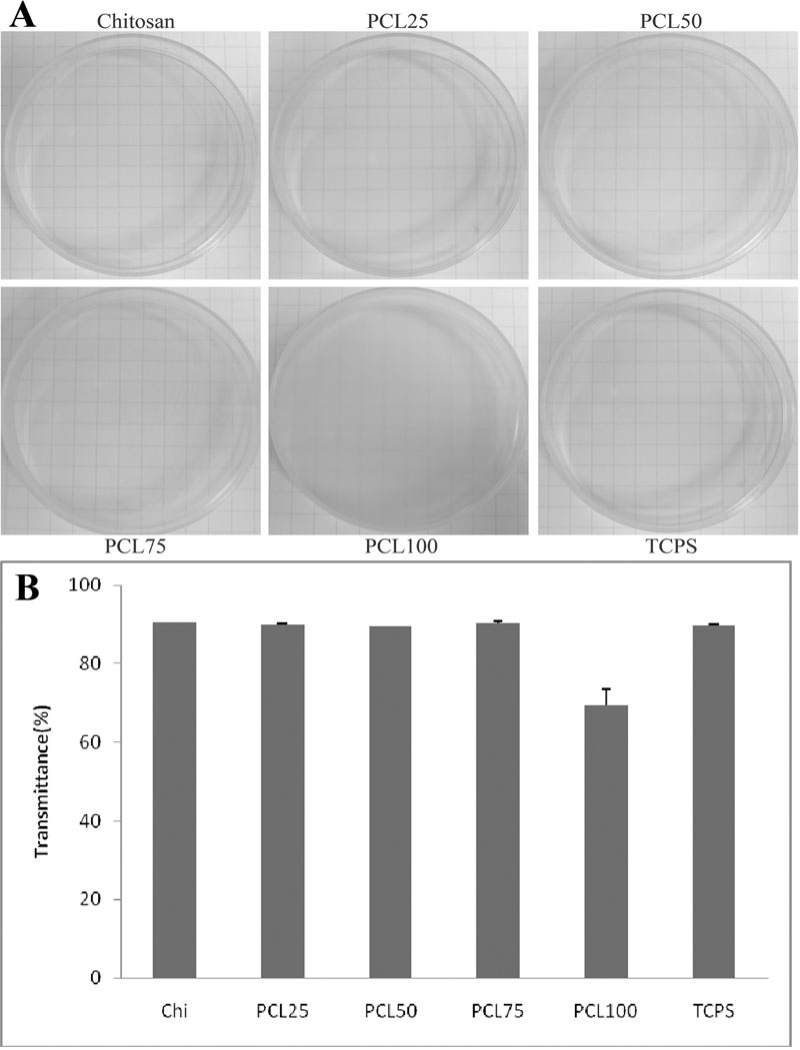
The transparency and light transmittance of the prepared culturing substrates. **A**: The lines of graph paper could be easily seen through chitosan, PCL 25, PCL 50, and PCL 75. As for PCL 100, it is remarkable that the prepared culturing substrate became opaque. **B**: The light transmittance decreased particularly through PCL 100.

The cornea is the outermost window in the visual pathway, optical transparency of implanted biomaterials is also important for vision. During CEC culture and transplantation, transparent membranes enable observation of cell behavior, healing process and signs of possible infection. Therefore, transparency of prepared membranes is significant for clinical applications [21]. The result is shown in Figure 1A. The graph paper was clearly observed through the empty TCPS. In the experimental group, the lines on the graph paper were easily viewed through chitosan, PCL 25, PCL 50, and PCL 75, indicating that chitosan blended with 25%, 50% and 75% of PCL still preserved transparency. However, the substratum became opaque in pure PCL (PCL 100) and the lines on the graph paper could hardly be traced, indicating that this sample was not transparent. Quantitatively, a similar diminishing transmittance through PCL 100 was measured when it was compared with chitosan and other blends (Figure 1B). Therefore, BCEC culture was not performed on PCL 100 in the following experiments.

### Cell morphology of cultured BCECs on the culturing substrates

**Figure 2:**
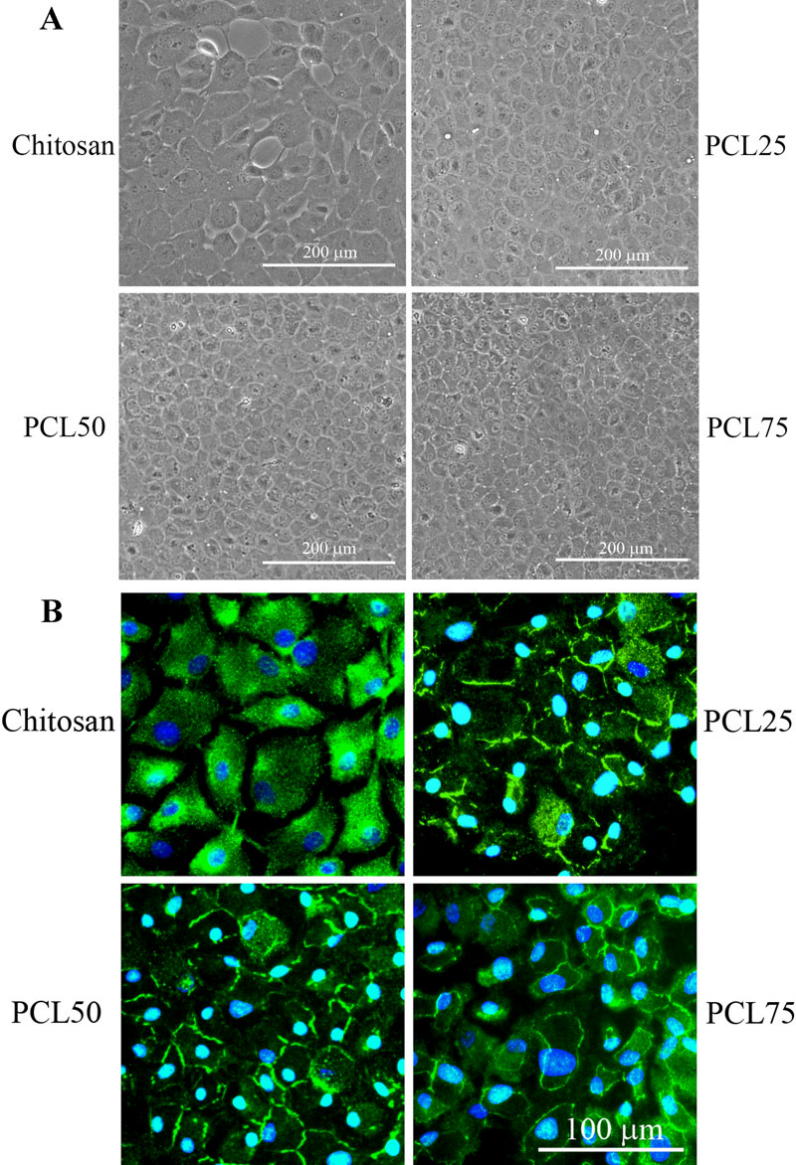
Cultivation of BCECs on chitosan and the blends. **A**: The morphological changes in BCECs cultured on chitosan, PCL 25, PCL 50, and PCL 75 after culture. At day 7, BCECs reached confluence in PCL 25, PCL 50 and PCL 75 groups. **B**: The expressions of ZO-1 (green) of BCECs cultured on chitosan, PCL 25, PCL 50, and PCL 75 after 7 days of incubation. Scale bar=100 µm.

Figure 2A illustrated BCEC morphology on the blends after 7 days by phase contrast microscope. BCECs adhered on chitosan but appear dispersed. On PCL blends (PCL 25, PCL 50, and PCL 75), BCECs spread well and reached confluence. Moreover, immunofluorescent staining of cultured BCECs on the culturing substrates showed expression of tight junction marker, ZO-1 protein, at the margin of cells. ZO-1 is a tight-junction-associated protein located in the CEC monolayer [19,20]. These findings confirmed the physiologic phenotypes of BCECs cultured on PCL 25, PCL 50, and PCL 75 substrates after 7 days of incubation (Figure 2B). With increasing PCL content, the expression patterns seem to become more obvious in PCL 50 and PCL 75. On the other hand, expression of ZO-1 in BCECs cultured on chitosan was not prominent at cell junction indicated that BCECs did not develop to the terminal stage morphology present in typical cells.

### Collagen type IV production

**Figure 3:**
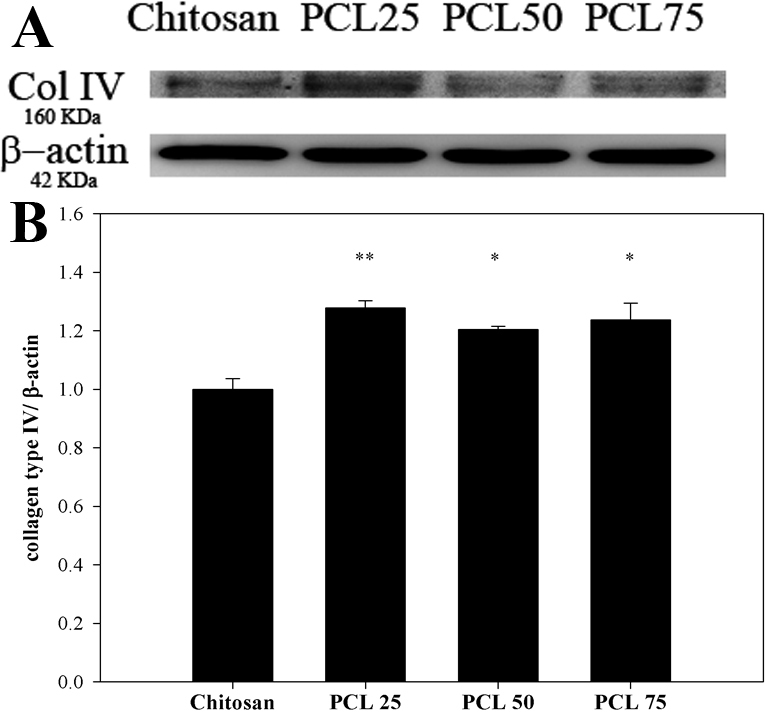
The effects of the blends on expression of collagen type IV in cultivated BCECs. **A**: Western blot analysis of anti- collagen type IV and anti-β-actin in BCECs cultured on chitosan, PCL 25, PCL 50, and PCL 75 after 7 days of incubation. Increased expression of type IV collagen, a corneal endothelial cell marker, with a molecular weight of 160 KDa was clearly observed in BCECs cultured on blends. **B**: The relative intensities of collagen type IV level were determined by band densitometry analysis. The ratio of the band intensities was expressed as a percentage of chitosan. Results were expressed as mean±SD from three independent experiments (*p<0.05 or **p<0.01).

To determine whether blends were able to stimulate BCECs increasing extracellular matrix (ECM) protein production, the amount of collagen type IV of BCECs cultured on chitosan, PCL 25, PCL 50, and PCL 75 after 7 days of incubation was assessed by western blot analysis. On all culturing substrates, expressions of collagen type IV were observed (Figure 3A). With normalization to the expression of β-actin, Figure 3B showed the production of collagen type IV by BCECs on blends was significantly higher than that on chitosan. By comparing with the amount of collagen type IV production on chitosan, the ratio on blends was 1.28 in PCL 25 (p=0.003), 1.20 in PCL 50 (p=0.04), and 1.24 in PCL 75 (p=0.02), respectively. These findings indicated that blends were presumably collagen type IV stimulants in BCECs. Furthermore, BCECs cultured on PCL25 had the highest production of collagen type IV in this study.

### The macroscopic features of blend membranes

**Figure 4:**
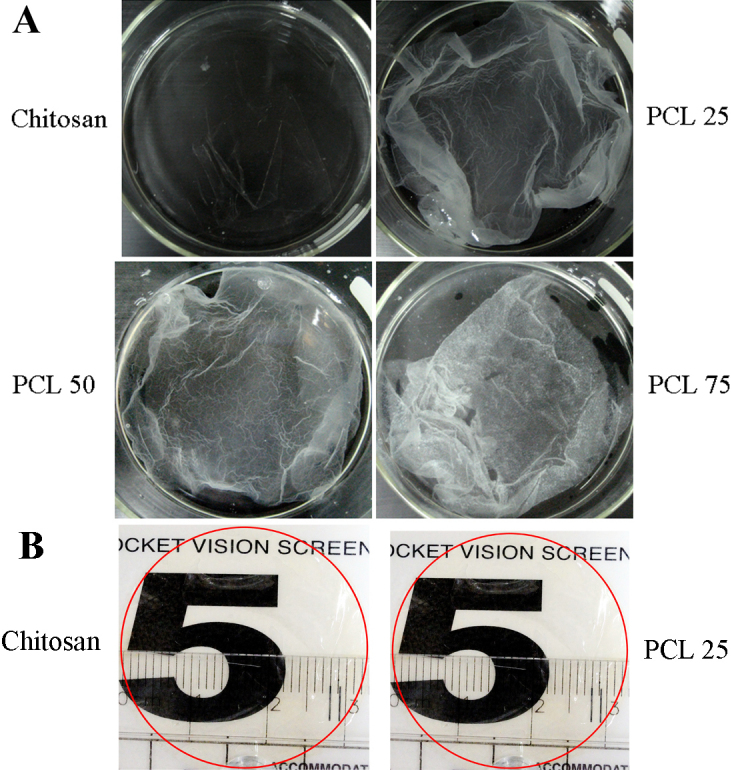
The appearance of the blend membranes formed by dry process. **A**: Macroscopic view of the blended membranes and placed in glass plate under wet conditions with PBS. **B**: The appearance of chitosan and PCL 25 membrane after cutting out in size of 6-well TCPS plate and placed on vision screen chart.

The mixture of chitosan and PCL polymer solutions were dried at 55 °C (near the PCL melting point) in the oven. Membranes, which formed and detached from glass plate, were highly homogeneous in structure and had a distinctive morphology. Avoided from splitting and dryness, prepared membranes were taken photographs by digital camera in 10 cm glass plate full under wet conditions with PBS (Figure 4A). Obviously, increased PCL component diminished the transparent character of chitosan membrane. These membranes were tried to cut in size to fit 24-well TCPS for culture. Nevertheless, PCL 50 and PCL 75 were fragile and with difficulty to fabricate carrier membranes. Only pure chitosan and PCL 25 (75 wt% of chitosan with 25 wt% of PCL) membranes had enough strength. Chitosan and PCL 25 membrane were then cut out in size of 6-well TCPS plate. Figure 4B illustrated the transparency of both membranes. The number “5” on the vision screen chart could be easily visualized.

### Cell morphology and phenotype expression of cultured BCECs on PCL 25 blend membrane

**Figure 5:**
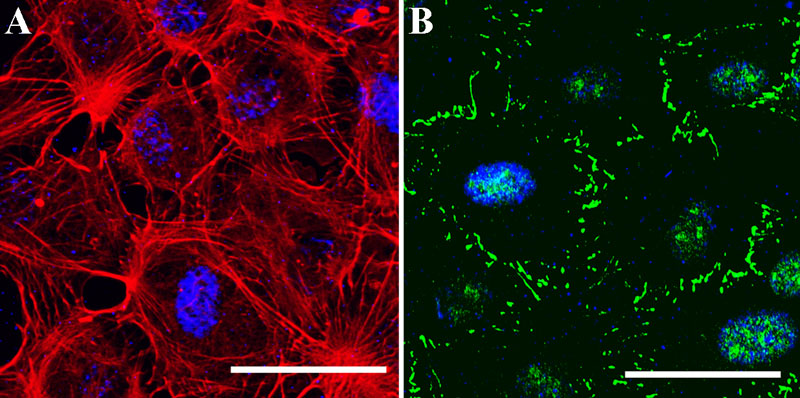
Immunohistochemistry of cultured BCECs on PCL 25 blend membrane. **A**: The expressions of cytoskeleton actin filament of BCECs at day 7 by confocal microscope. **B**: Correspondingly, the expressions of ZO-1 (green) at the margin of cells after 7 days. Scale bar=50 µm.

Although chitosan membrane could be prepared, the growth and phenotype expression of BCECs were not superior to those on blends in the previous experiments. We only cultivated BCECs on PCL 25 blend membrane. By means of confocal microscope, Figure 5A demonstrated BCEC cytoskeleton with well spread pattern on PCL 25 blend membrane at day 7. In addition, ZO-1 protein expression was confirmed at the margin of cells in Figure 5B. These findings might also imply that this carrier material did not cause cytotoxicity.

## Discussion

For biodegradable biomaterials, chitosan is a linear polysaccharide with a changeable number of randomly placed N-acetyl-glucosamine groups. It is formed from fully or partially deacetylated chitin, which is the second-most abundant polymer in nature. Chitosan has been extensively applied in tissue engineering because of its biocompatibility, biodegradability, non-antigenic effect, wound healing properties, and low costs [22]. It can also promote ECM fabrication [23,24]. Despite these advantages, its current usage in tissue engineering is limited mainly because of low strength and incomplete understanding of cellular interactions with chitosan. Nevertheless, several approaches have been taken to overcome the restrictions of chitosan, including graft polymerization [25,26] and blending [27,28]. Surprisingly, the biologic, mechanical or degradation properties of polymer blends have revealed promising results in comparison to that on the separate culturing substratum. Alternatively, PCL, a flexible synthetic polymer with low melting point (60 °C), allows for easy processing and is therefore a suitable selection for blending. Additionally, it is biodegradable and biocompatible polyester with excellent tensile properties [17]. Nevertheless, a major drawback of PCL is reduced bioregulatory activity primarily in tissue engineering; this is because of its hydrophobic nature and slow rate of biodegradation. However, the flexibility of PCL in blending with other polymers allows the adjustment of its properties to overcome its drawbacks [17].

According to Williams [29], a biomaterial is “*a substance that has been engineered to take a form which, alone or as part of a complex system, is used to direct, by control of interactions with components of living systems, the course of any therapeutic or diagnostic procedure, in human or veterinary medicine*.” Native corneal endothelium is a densely packed monolayer of regular hexagonal cells boarding the posterior aspect of the cornea [4]. From the perspective of CEC tissue engineering, a successful biomaterial should support cells to adhere, proliferate, and execute their physiologic function. Both chitosan and PCL have been approved by FDA as biodegradable biomaterials. Without complex chemical modification, blending hydrophilic chitosan and hydrophobic PCL can be achieved and represented as a good model for two semicrystalline polymers [17,18]. On chitosan and PCL blends, cells can modulate gene expression and produce collagen through cell shape changes [18]. Hence, based on different adhesion effects on chitosan and PCL, the present study is focused on the behavior of CECs on chitosan and PCL blends.

In the initial part of this test, the BCECs were cultured on a series of chitosan and PCL blends. Appropriate transparency cannot be maintained on pure PCL (Figure 1A,B). As for transmittance, the transparency of the biomaterial membranes is advantageous for clinical application. Although chitosan could not support CEC adhesion entirely, polymer blending can effectively change the properties of a biomaterial. Nevertheless, due to poor transparency, PCL 100 was not used in the subsequent experiments. For cell observation during culture, pure chitosan, PCL 25, PCL 50 and PCL 75 substrates were directly observed under inverse phase contrast microscope. On chitosan and PCL blends, BCECs reached confluence in 7 days (Figure 2A). The results obtained from chitosan substrate were inconclusive. In addition to cell morphology, expression of tight junction ZO-1 was used to confirm the cultured BCECs without changing their phenotype on the substrata during culture. Likewise, the expression patterns of ZO-1 were not observed in cultured BCECs on chitosan, indicating BECEs did not develop to its mature morphology (Figure 2B). In contrast, when BCECs were cultured on PCL 25, PCL 50, and PCL 75, the expression of tight junction ZO-1 was well developed, resembling ideal physiologic phenotypes [19,20]. In the long term cellular responses on biomaterials, endogenous ECM proteins synthesized by cells are crucial for cell activities. For instance, CECs can synthesize collagen type IV which is deposited and specifically found in their basement membrane. Also, Tseng et al. [14] have reported that specific ECM proteins, collagen type IV, can be produced by cultured CECs when they reach confluence. In agreement with the concept that the culture system used for tissue engineering should be bioactive, the biomaterial which is capable of simulating endogenous ECM production is always preferable [14]. As shown in Figure 3, western blot analysis demonstrated that the amount of collagen type IV production of BCECs cultured on blends was greater than that on chitosan. On the other hand, we performed western blotting for type I collagen, which represented a fibrosis marker of CECs according to previous studies [30]. The expressions of collagen I demonstrated extremely few on the blends (data not shown). For that reason, TGF-beta/Smad signals may be related to corneal endothelial fibrosis. The cell activity and signaling between CECs and blends will be worthy further surveyed. Therefore, these results further assure that behaviors of BCECs are strongly dependent on the surface characteristics of biomaterials.

In the subsequent part of this investigation, we made effort in preparation of blend membrane from a single phase solution. Since chitosan has relative low break stress and elastic modulus, blending with PCL would provide an opportunity to create a material with proper tensile properties. The membranes fabricated from homogenous solution with different proportions of chitosan and PCL by dry process were shown in Figure 4A. Nevertheless, homogenous solution of PCL 50 and PCL 75 produced very brittle membranes. This could be explained by the highly crystalline nature of PCL. For potential application in corneal endothelial transplantation, membranes had to be cut out in desired size. Chitosan and PCL 25 blend membrane were successfully generated with optical clarity after drying (Figure 4B). We cultivated BCECs on PCL 25 membrane because they could not adhere and proliferate well on chitosan in the previous experiments. Captured images by confocal microscopic system in Figure 5, cultured BCECs on PCL 25 blend membrane spread out well and displayed physiologic phenotype of ZO-1. These results suggested that PCL 25 blend membrane was able to support the function of the BCECs in vitro. The major findings in these experiments illustrated that blending chitosan with PCL successfully allowed cell adherence, improved phenotypic expressions, and maintained transparency. In a specific proportion, blend membrane was fabricated to be a scaffold and carrier with sufficient mechanical strength. As compared to manufacture of other carriers, our PCL blend membrane could be prepared easily without complex processing. Since our final goal is to develop carrier sheet, the establishment of animal model for corneal endothelial transplantation may be required in the future studies.

### 

#### Conclusions

In the first part of experiment, hydrophilic chitosan was blended successfully with hydrophobic PCL in homogenous solutions without complex chemical modification. Transparency could be maintained in chitosan and PCL blends as well. Furthermore, BCECs reached confluence and expressed tight junction and ECM proteins on blends. In the later part of research, only 75:25 (chitosan: PCL) could make blend membrane with enough strength for scaffold and carrier in culture. On this blend membrane, BCECs could expand and grow well. Therefore, novel chitosan and PCL blend membrane to be a biomaterial may provide the opportunity for surveying further transplantation of bioengineered corneal endothelium.
